# Educational Interventions for Professional Identity Formation in Undergraduate Medical Education: A Scoping Review

**DOI:** 10.7759/cureus.117860

**Published:** 2026-10-13

**Authors:** Dinesh Kumar Narayanasamy, Divyashanthi Chellathambi Malathi, Nilakantan Ananthakrishnan

**Affiliations:** 1 Pediatrics, Jawaharlal Institute of Postgraduate Medical Education and Research, Karaikal, IND; 2 Master of Health Professions Education (MHPE), Institute of Health Professions Education, Sri Balaji Vidyapeeth University, Puducherry, IND; 3 Pharmacology, Jawaharlal Institute of Postgraduate Medical Education and Research, Karaikal, IND; 4 Surgery and Health Professions Education, Institute of Health Professions Education, Sri Balaji Vidyapeeth University, Puducherry, IND

**Keywords:** attitude ethics and communication (aetcom), competency-based medical education (cbme), educational interventions, ethics, low- and middle-income countries, professional identity formation, scoping review, undergraduate medical education

## Abstract

Professional identity formation (PIF) is the process through which medical students integrate professional values, norms, and behaviours into their developing professional identity. Although PIF is increasingly recognised as an educational goal in undergraduate medical education (UGME), intervention research remains heterogeneous in educational approaches, theoretical foundations, outcome measurement, and geographic distribution. This scoping review mapped educational interventions designed to support PIF in UGME, their theoretical foundations, outcome and measurement approaches, and geographic and income-group distribution. PubMed, Embase, Scopus, and ERIC were searched for English-language studies published from 2010 to 2025, supplemented by grey literature and citation searching. Studies addressing PIF-focused educational interventions among undergraduate medical students were eligible. Two reviewers independently screened records, with disagreements resolved through discussion or adjudication by a third reviewer. Data were charted using a piloted form and narratively synthesised using an inductively derived seven-category intervention taxonomy. Fifty-two studies from 21 countries were included. Interventions comprised clinical exposure (n=13, 25.0%), humanities/arts (n=11, 21.2%), reflective/narrative approaches (n=9, 17.3%), mentorship (n=7, 13.5%), mixed interventions (n=5, 9.6%), simulation (n=4, 7.7%), and peer learning (n=3, 5.8%). Identity development was the predominant outcome (n=32, 61.5%). Theoretical or conceptual frameworks were reported in 44 studies (84.6%), whereas eight (15.4%) reported none. Validated instruments were used in only three studies (5.8%). Forty-nine studies (94.2%) originated from high-income countries, two (3.8%) from lower-middle-income countries, and one (1.9%) from an upper-middle-income country. Four studies reported null or near-null effects. PIF intervention research remains theoretically heterogeneous, measurement-limited, and geographically concentrated. Greater use of validated, culturally responsive measures and longitudinal, context-sensitive research, particularly in low- and middle-income settings, is needed.

## Introduction and background

Professional identity formation (PIF) is the ongoing process through which medical students integrate the values, behaviours, and ways of thinking of the profession into their developing sense of self [1,2]. Medical education reform has recognised that technical competence cannot be separated from this process of professional development, while disruptions in professional identity have been associated with professionalism lapses, patient-safety concerns, and discrepancies between espoused and enacted professional values [3-5]. PIF has therefore evolved from an implicit consequence of clinical training into an explicit goal of undergraduate medical education (UGME), building on sociological concepts of professional socialisation [6]. Cruess et al. positioned professional identity above the “knows”, “knows how”, and “shows how” levels of Miller’s pyramid [7], highlighting the distinction between interventions that influence observable performance and those that shape the identity underlying professional behaviour.

PIF is multidimensional and requires complementary theoretical perspectives [8,9]. The Ring Theory of Personhood emphasises individual, relational, and societal influences, while communities of practice and socialisation theories focus on participation, relationships, and the formal, informal, and hidden curricula [10-12]. Transformative learning further highlights the role of critical reflection following disorienting experiences [13,14].

Four previous reviews have advanced understanding of PIF but leave important gaps. Mount et al. identified a predominantly individualist, reflection-oriented paradigm across 43 studies but did not develop an intervention taxonomy or examine geographic distribution [15]. Sarraf-Yazdi et al. mapped PIF across 76 articles without systematically classifying interventions or theoretical frameworks [16]; their subsequent conceptual synthesis included 204 articles across the medical education continuum but did not specifically map UGME interventions or their theoretical grounding [17]. Toh et al. examined relational approaches, including mentoring, supervision, coaching, and teaching, whereas Tabatabaei et al. highlighted the limited availability of psychometrically robust PIF measures [18,19]. Collectively, these reviews do not provide an integrated UGME-specific map of intervention types, theoretical foundations, outcome measurement, and geographic distribution.

This scoping review, therefore, aimed to map educational interventions designed to support PIF in UGME, with the following research questions (RQs): (i) RQ1: What educational interventions have been designed and evaluated to support PIF among undergraduate medical students? (ii) RQ2: What theoretical frameworks underpin these interventions, and how explicitly are they applied? (iii) RQ3: How is PIF measured, and what outcomes are reported? (iv) RQ4: How are PIF interventions distributed across geographic and income-group contexts, particularly in low- and middle-income countries (LMICs)?

## Review

Methods

Study Design and Reporting Framework

This study was conducted as a scoping review rather than a systematic review, consistent with guidance that scoping reviews are the appropriate design when the aim is to characterise the extent, range, and nature of evidence in a field marked by conceptual and methodological heterogeneity [20]. This description fits PIF intervention research, where designs, outcome measures, theoretical frameworks, and intervention types vary widely. The review was conducted and reported according to the five-stage framework of Arksey and O’Malley [21], as refined by Levac et al. [22], and operationalised using JBI guidance for scoping reviews [23]. The review is reported according to the PRISMA-ScR checklist (see Appendix) [24].

Protocol and Registration

The protocol was prospectively registered on the Open Science Framework (Registration DOI: 10.17605/OSF.IO/E9G8B), specifying eligibility criteria, the data extraction template, and the planned analytical approach in advance of screening.

Eligibility Criteria

Eligibility was defined using the Population-Concept-Context (PCC) framework recommended for scoping reviews.

Population: Undergraduate medical students enrolled in MBBS or an equivalent undergraduate medical degree programme were the focus. Postgraduate trainees and non-undergraduate populations were excluded.

Concept: Educational interventions were defined as deliberately designed pedagogic activities, programmes, or curriculum elements with an explicit educational objective and evaluated for their effect on PIF or a closely related construct, with PIF identified as a primary or clearly articulated secondary outcome. Observational, exploratory, or conceptual studies without a designed educational intervention were excluded.

Context: UGME in any country, without geographic restriction, was considered to permit analysis of geographic and income-group distribution.

Studies published in English between 1 January 2010 and 31 December 2025 were eligible. The 2010 start date was selected based on the emergence of identity formation as a construct distinct from competency [3], while the 2025 end date extended coverage beyond the most recent critical review [15].

Information Sources and Search Strategy

Four bibliographic databases were searched systematically: PubMed (MEDLINE), Embase, Scopus, and Education Resources Information Centre (ERIC). These databases provided coverage across biomedical, multidisciplinary, and education-focused literature. Google Scholar was additionally searched for the first 100 results to identify relevant grey literature on 29 April 2026. Backward and forward citation searching was conducted from two key reviews [16,17].

The search strategy combined terms for population, concept, and context using Boolean operators, truncation, and controlled vocabulary, including MeSH terms in PubMed. Full search strings for each database, including the Scopus string, are provided in Table 1.

**Table 1:** Search Strategies for the Included Databases Search strategies were developed by the authors and adapted for each database using database-specific controlled vocabulary, keywords, Boolean operators, and truncation. Full search strategies for PubMed, Embase, Scopus, and ERIC are provided. Google Scholar was searched separately for supplementary grey literature.

PubMed (MEDLINE)
("professional identity formation"[tiab] OR "professional identity"[tiab] OR "physician identity"[tiab]) AND ("intervention"[tiab] OR "curriculum"[tiab] OR "programme"[tiab] OR "program"[tiab] OR "mentorship"[tiab] OR "mentoring"[tiab] OR "reflective"[tiab] OR "portfolio"[tiab] OR "narrative"[tiab] OR "role model"[tiab] OR "role modelling"[tiab] OR "experiential"[tiab] OR "community placement"[tiab] OR "service learning"[tiab] OR "simulation"[tiab] OR "humanities"[tiab] OR "arts"[tiab] OR "peer learning"[tiab]) AND ("medical student"[tiab] OR "undergraduate medical"[tiab] OR "MBBS"[tiab] OR "medical school"[tiab] OR "Students, Medical"[MeSH]) AND ("2010/01/01"[Date - Publication] : "2025/12/31"[Date - Publication])
EMBASE
('professional identity formation':ti,ab OR 'professional identity':ti,ab OR 'physician identity':ti,ab) AND ('intervention':ti,ab OR 'curriculum':ti,ab OR 'programme':ti,ab OR 'program':ti,ab OR 'mentorship':ti,ab OR 'mentoring':ti,ab OR 'reflective':ti,ab OR 'portfolio':ti,ab OR 'narrative':ti,ab OR 'role model':ti,ab OR 'role modelling':ti,ab OR 'experiential learning':ti,ab OR 'community placement':ti,ab OR 'service learning':ti,ab OR 'simulation':ti,ab OR 'humanities':ti,ab OR 'arts':ti,ab OR 'peer learning':ti,ab) AND ('medical student'/exp OR 'medical student':ti,ab OR 'undergraduate medical':ti,ab OR 'mbbs':ti,ab OR 'medical school':ti,ab) AND 2010-2025/py
SCOPUS
TITLE-ABS( "professional identity formation" OR "professional identity" OR "physician identity" ) AND TITLE-ABS( intervention OR curriculum OR programme OR program OR mentorship OR mentoring OR reflective OR reflection OR portfolio OR narrative OR "role model" OR "role modelling" OR simulation OR "experiential learning" OR "community placement" OR "service learning" OR "peer learning" OR arts OR humanities ) AND TITLE-ABS( "medical student" OR "medical students" OR "undergraduate medical" OR "medical school" OR MBBS ) Filters: - Year: 2010–2025 Language: English
Education Resources Information Centre (ERIC)
("Professional Identity" OR "professional identity formation" OR "physician identity") AND ("Curriculum Development" OR "Educational Methods" OR "Teaching Methods" OR "Mentoring" OR "Reflective Practice" OR "Experiential Learning" OR "Simulation" OR "Role Models" OR "Portfolio Assessment" OR "Service Learning" OR "Peer Teaching" OR "Humanities" OR intervention* OR program* OR programme* OR curriculum OR mentoring OR reflective OR portfolio OR narrative OR "role model*" OR simulation OR "peer learning" OR "service learning") AND ("Medical Students" OR "Undergraduate Students" OR "Medical Education" OR "medical student*" OR "undergraduate medical" OR MBBS OR "medical school*") AND (2010-2025)

Selection of Sources of Evidence

Citations of interest were imported into Zotero for deduplication and subsequently transferred to Rayyan for screening [25]. We carried out a two-stage screening process consisting of title and abstract screening followed by full-text review. Initially, titles and abstracts were screened against the pre-established eligibility criteria. Subsequently, a comprehensive full-text review was performed for all potentially eligible articles to confirm their suitability for final inclusion. Two reviewers (DKN and DS) independently screened the titles and abstracts and subsequently the full texts against the eligibility criteria. Disagreements at both stages were resolved through discussion, with a third reviewer (AK) adjudicating any disagreement that could not be resolved by consensus. Reasons for exclusion at the full-text stage were recorded and are reported in the PRISMA-ScR flow diagram.

Data Charting Process and Data Items

A piloted data charting form was used to extract relevant information from the included studies. The extracted variables comprised author and year, country, World Bank income classification, programme type, study design, sample size, year of training, setting, intervention type and description, duration, delivery mode, theoretical framework, PIF focus, outcome domain, measurement method and instruments, key findings, facilitators, barriers, study limitations, and explicit reporting of intersectionality. Data charting was independently performed by two reviewers, with discrepancies resolved through discussion.

Following data charting, interventions were inductively classified according to their predominant pedagogic mechanism. Seven categories emerged: clinical exposure, introduction of humanities/arts, reflective/narrative approaches, mentorship, simulation, peer learning, and mixed interventions. Mixed interventions comprised studies incorporating two or more major pedagogic modalities without a clearly dominant component. Classification was based on the predominant educational activity described by the study authors rather than secondary components such as reflection, mentorship, or peer discussion.

Analysis and Synthesis of Data

Data were analysed descriptively and synthesised narratively. Descriptive analysis characterised the distribution of included studies according to intervention type, study design, geographic and income-group context, theoretical framework, outcome domain, and measurement approach. Qualitative findings on facilitators, barriers, and reported intervention effects were synthesised narratively to identify recurring patterns across studies. Consistent with the purpose of a scoping review and JBI guidance [23], formal methodological quality appraisal was not undertaken.

Ethical Considerations

Formal institutional ethical approval was not required because this study synthesised secondary data from previously published literature and did not involve human participants or the collection of primary data. Research integrity and publication ethics were maintained through accurate representation of the included evidence and meticulous citation of all primary sources.

Results

Study Selection

Searches of PubMed, Embase, Scopus, and ERIC, supplemented by grey-literature and backward and forward citation searching, identified 2,187 records. After removal of 855 duplicates, 1,332 records underwent title and abstract screening. Of these, 977 were excluded and two could not be retrieved. A total of 353 full-text articles were assessed for eligibility, of which 301 were excluded. Fifty-two studies met the eligibility criteria and were included in the final synthesis. The selection process is presented in the PRISMA-ScR flow diagram (Figure 1).

**Figure 1:**
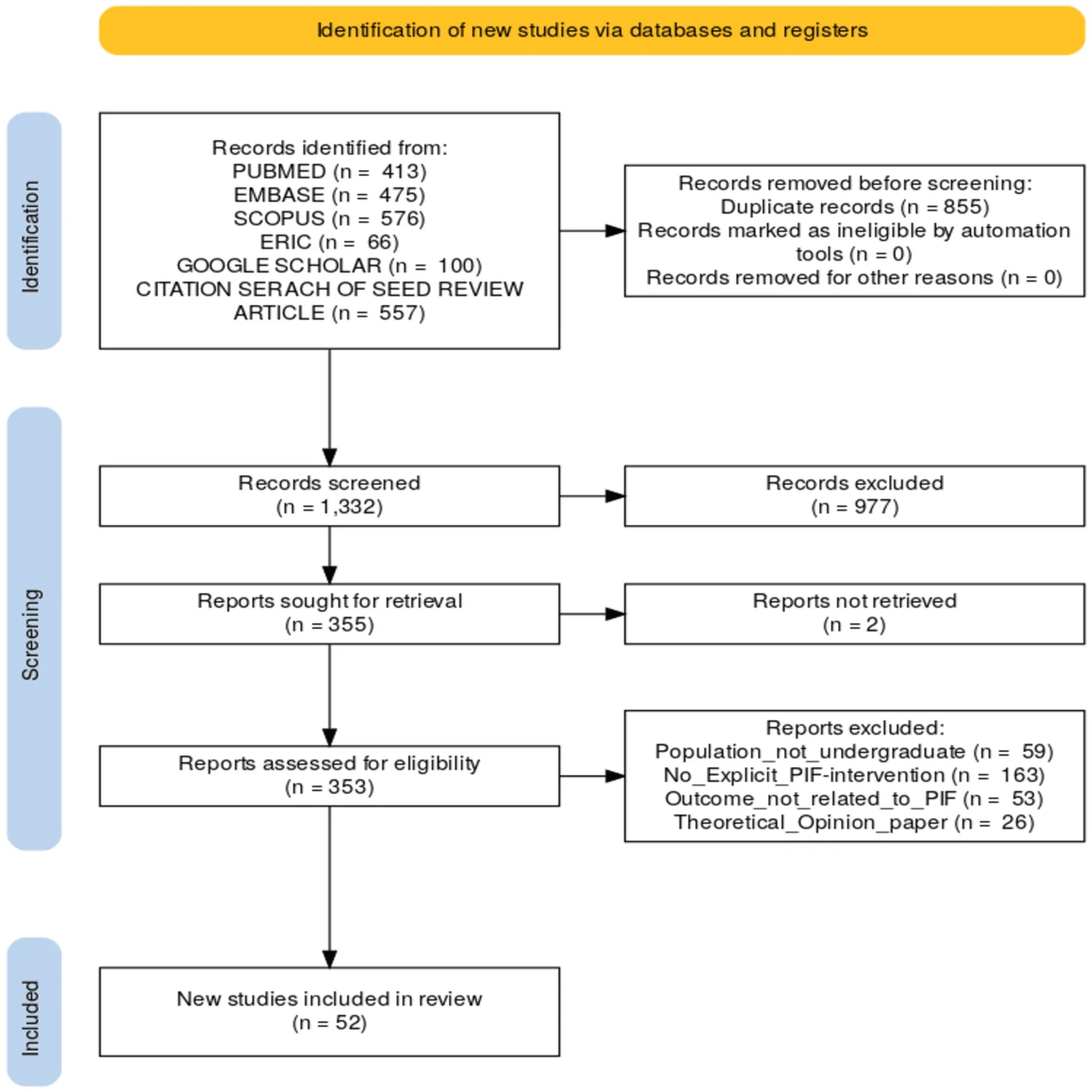
PRISMA Flow Diagram of the Study Selection Process The diagram shows the identification, screening, eligibility assessment, and inclusion of records in the scoping review. PRISMA, Preferred Reporting Items for Systematic Reviews and Meta-Analyses Extension for Scoping Reviews; PIF, professional identity formation

Characteristics of Included Studies

The 52 included studies were published between 2010 and 2025 and represented 21 countries or multi-country settings. Ten studies were published before 2020, while 42 (80.8%) were published from 2020 onwards, including 12 studies published in 2025. Sample sizes ranged from 8 to 1,096 participants. Study design, curricular phase, delivery mode, and PIF focus are summarised in Table 2, with individual study characteristics presented in Table 3.

**Table 2:** Characteristics of Included Studies (n=52) Authors' analysis of data extracted from the 52 included studies. The curricular phase was classified according to the stage of the undergraduate medical curriculum in which the intervention was delivered. PIF, professional identity formation

Characteristic	Number of studies, n (%)
Study design	
Qualitative	19 (36.5)
Mixed methods	15 (28.8)
Programme evaluation	10 (19.2)
Quantitative	8 (15.4)
Curricular phase	
Preclinical	22 (42.3)
Clinical	17 (32.7)
Both preclinical and clinical	12 (23.1)
Not reported	1 (1.9)
Delivery mode	
Group	27 (51.9)
Individual	14 (26.9)
Blended	11 (21.2)
PIF focus	
Primary outcome	42 (80.8)
Secondary outcome	10 (19.2)

**Table 3:** Complete Characteristics of the Included Study (n=52) Authors' analysis of data extracted from the 52 included studies. Study-level characteristics were extracted using a piloted data charting form. Income group was classified according to World Bank income categories. PIF, professional identity formation; HIC, high-income country; LMIC, low-income and middle-income country; UMIC, upper-middle-income country

S No	Author, year, reference	Country	World Bank income	Study design	Setting	Intervention type	Duration	Delivery mode	PIF focus	Outcome type	Measurement method
1	Altmann et al. (2025) [26]	Austria	HIC	Quantitative	Clinical	Peer Learning	90 minutes (single session)	Group	Primary outcome	Belonging	Mixed
2	Beck et al. (2015) [27]	USA	HIC	Qualitative	Preclinical	Clinical Exposure	1 week	Group	Primary outcome	Identity Development	Qualitative
3	Kanwar et al. (2024) [28]	India	LMIC	Quantitative	Preclinical	Clinical Exposure	NR (semester-length implied)	Group	Secondary outcome	Professional Values	Non-validated scale
4	Schei et al. (2019) [29]	Norway	HIC	Qualitative	Preclinical	Clinical Exposure	6 months (40-hour program)	Group	Primary outcome	Identity Development	Qualitative
5	Chrisman-Khawam et al. (2020) [30]	USA	HIC	Qualitative	Preclinical	Clinical Exposure	Variable (weekly clinics over 2 academic years)	Group	Primary outcome	Empathy	Qualitative
6	Kovaric and Luk (2025) [31]	USA	HIC	Qualitative	Clinical	Clinical Exposure	1 year (academic year)	Group	Primary outcome	Empathy	Qualitative
7	Busey et al. (2025) [32]	USA	HIC	Programme Evaluation	Both	Clinical Exposure	4 years (longitudinal)	Blended	Primary outcome	Identity Development	Non-validated scale
8	Lambdin et al. (2023) [33]	USA	HIC	Programme Evaluation	Preclinical	Clinical Exposure	3 immersions of 3 weeks each	Blended	Primary outcome	Identity Development	Mixed
9	Jeon et al. (2022) [34]	South Korea	HIC	Qualitative	Clinical	Clinical Exposure	3 weeks	Group	Secondary outcome	Professional Values	Qualitative
10	Adams et al. (2020) [35]	USA	HIC	Qualitative	Clinical	Clinical Exposure	11 months	Individual	Primary outcome	Identity Development	Qualitative
11	Ong et al. (2023) [36]	Singapore	HIC	Mixed	Preclinical	Clinical Exposure	Duration of anatomy course (NR)	Group	Secondary outcome	Identity Development	Mixed
12	Ahmad et al. (2018) [37]	Malaysia	HIC	Qualitative	Clinical	Clinical Exposure	NR (embedded in curriculum)	Group	Primary outcome	Identity Development	Qualitative
13	Darici et al. (2022) [38]	Germany	HIC	Mixed	Preclinical	Clinical Exposure	4-day block course	Group	Primary outcome	Identity Development	Mixed
14	Ogier et al. (2025) [39]	UK	HIC	Qualitative	Preclinical	Clinical Exposure	2 years (2 modules)	Blended	Primary outcome	Identity Development	Qualitative
15	Lawrence et al. (2020) [40]	USA	HIC	Quantitative	Preclinical	Humanities/Arts	5 sessions (longitudinal within 1 semester)	Group	Primary outcome	Empathy	Non-validated scale
16	Reis and Wald (2015) [41]	Israel	HIC	Programme Evaluation	Preclinical	Humanities/Arts	20 months	Blended	Primary outcome	Professional Values	NR
17	Li et al. (2025) [42]	USA	HIC	Qualitative	Both	Humanities/Arts	7-day trip + pre/post curriculum	Group	Primary outcome	Professional Values	Qualitative
18	Riesen et al. (2023) [43]	Germany	HIC	Qualitative	Preclinical	Humanities/Arts	NR (multi-module over semester)	Blended	Primary outcome	Professional Values	Qualitative
19	Stouffer et al. (2021) [44]	USA	HIC	Mixed	Clinical	Humanities/Arts	1 week (40 hrs/week)	Group	Primary outcome	Identity Development	Mixed
20	Aluri et al. (2023) [45]	USA	HIC	Qualitative	Clinical	Humanities/Arts	1 week	Group	Primary outcome	Identity Development	Qualitative
21	Green (2015) [46]	USA	HIC	Programme Evaluation	Clinical	Humanities/Arts	4 weeks	Group	Primary outcome	Identity Development	Mixed
22	Tackett et al. (2023) [47]	USA	HIC	Mixed	Clinical	Humanities/Arts	4 weeks (17 sessions)	Group	Primary outcome	Identity Development	Mixed
23	Kagan et al. (2022) [48]	USA	HIC	Qualitative	Clinical	Humanities/Arts	6 sessions (~6 months)	Group	Secondary outcome	Identity Development	Qualitative
24	Shapiro et al. (2018) [49]	USA	HIC	Mixed	Preclinical	Humanities/Arts	Single session (wellness retreat)	Group	Primary outcome	Identity Development	Mixed
25	Shapiro et al. (2021) [50]	USA	HIC	Mixed	Both	Humanities/Arts	Single session each (NR)	Group	Primary outcome	Identity Development	Mixed
26	Trippenzee et al. (2025) [51]	Netherlands	HIC	Programme Evaluation	Preclinical	Reflective/Narrative	2 hours (single session)	Group	Primary outcome	Identity Development	Non-validated scale
27	Shiozawa et al. (2020) [52]	Germany	HIC	Qualitative	Preclinical	Reflective/Narrative	One academic term (dissection course)	Blended	Primary outcome	Professional Values	Qualitative
28	Felaza et al. (2025) [53]	Indonesia	HIC	Quantitative	Clinical	Reflective/Narrative	10 months (May 2022–Mar 2023)	Individual	Secondary outcome	Identity Development	Non-validated scale
29	Arthur et al. (2025) [54]	USA	HIC	Qualitative	Preclinical	Reflective/Narrative	Duration of dissection course (NR)	Individual	Primary outcome	Identity Development	Qualitative
30	Hilgeman et al. (2025) [55]	USA	HIC	Mixed	Clinical	Reflective/Narrative	4 weeks (1-month clerkship)	Individual	Primary outcome	Empathy	Mixed
31	Matsuyama et al. (2021) [56]	Japan	HIC	Quantitative	Preclinical	Reflective/Narrative	7 months	Individual	Primary outcome	Identity Development	Validated Instrument
32	Kneese et al. (2020) [57]	USA	HIC	Mixed	Preclinical	Reflective/Narrative	Academic year	Individual	Primary outcome	Identity Development	Mixed
33	Olivares-Olivares et al. (2024) [58]	Mexico, Colombia, Chile	UMIC	Quantitative	Clinical	Reflective/Narrative	5-week pilot + implementation	Individual	Secondary outcome	Identity Development	Non-validated scale
34	Baseer et al. (2024) [59]	Pakistan	LMIC	Quantitative	Clinical	Reflective/Narrative	3 weeks	Individual	Primary outcome	Empathy	Validated Instrument
35	Kalén et al. (2010) [60]	Sweden	HIC	Mixed	Clinical	Mentorship	2 years	Individual	Secondary outcome	Identity Development	Mixed
36	Parsons et al. (2021) [61]	USA	HIC	Programme Evaluation	Both	Mentorship	4 years (longitudinal)	Blended	Primary outcome	Identity Development	NR
37	Kline et al. (2020) [62]	Canada	HIC	Qualitative	Preclinical	Mentorship	2 years (longitudinal preclinical elective)	Individual	Primary outcome	Professional Values	Qualitative
38	Dixon et al. (2022) [63]	USA	HIC	Mixed	Clinical	Mentorship	3 weeks	Individual	Primary outcome	Identity Development	Mixed
39	Chen et al. (2021) [64]	USA	HIC	Programme Evaluation	Both	Mentorship	Ongoing (longitudinal platform)	Individual	Secondary outcome	Belonging	Non-validated scale
40	Nguyen et al. (2025) [65]	Vietnam	HIC	Mixed	Both	Mentorship	Longitudinal (multi-year)	Individual	Primary outcome	Identity Development	Mixed
41	Krishna et al. (2023) [66]	Singapore	HIC	Qualitative	Both	Mentorship	Longitudinal (program duration NR)	Individual	Primary outcome	Identity Development	Qualitative
42	Désilets et al. (2022) [67]	Canada	HIC	Programme Evaluation	Preclinical	Mixed	4 years (longitudinal)	Blended	Primary outcome	Identity Development	Non-validated scale
43	Inayat et al. (2023) [68]	Canada	HIC	Mixed	Preclinical	Mixed	9 months	Blended	Primary outcome	Behaviour/Professionalism	Mixed
44	Findyartini and Sudarsono (2018) [69]	Indonesia	HIC	Qualitative	Preclinical	Mixed	6 weeks	Blended	Secondary outcome	Behaviour/Professionalism	Qualitative
45	Buck et al. (2021) [70]	USA	HIC	Programme Evaluation	Both	Mixed	4 years (longitudinal)	Blended	Primary outcome	Identity Development	Non-validated scale
46	Byrne et al. (2025) [71]	Ireland	HIC	Mixed	Preclinical	Mixed	Single session each (back-to-back)	Group	Primary outcome	Behaviour/Professionalism	Mixed
47	Feldman et al. (2022) [72]	USA	HIC	Mixed	Preclinical	Simulation	45 minutes (single session)	Group	Primary outcome	Identity Development	Mixed
48	Tien et al. (2019) [73]	USA	HIC	Mixed	Clinical	Simulation	Single session	Group	Primary outcome	Identity Development	Mixed
49	Burford et al. (2020) [74]	UK	HIC	Quantitative	Both	Simulation	Single session (NR duration)	Group	Secondary outcome	Belonging	Validated Instrument
50	De Grasset et al. (2018) [75]	Switzerland	HIC	Qualitative	Clinical	Simulation	6-month faculty development program	Group	Primary outcome	Belonging	Qualitative
51	Klitgaard et al. (2025) [76]	Denmark	HIC	Qualitative	Clinical	Peer Learning	NR (during clinical rotations)	Group	Primary outcome	Belonging	Qualitative
52	Drossard and Härtl (2024) [77]	Germany	HIC	Programme Evaluation	Preclinical	Peer Learning	1 semester (pilot); ongoing in-person	Group	Primary outcome	Identity Development	Non-validated scale

Intervention Types and Characteristics

Seven intervention categories were identified, including peer learning approaches such as interactive interprofessional learning [26], and are shown in Table 4.

**Table 4:** Classification of Educational Interventions for Professional Identity Formation (n=52) Authors' analysis of data extracted from the 52 included studies. Categories were assigned according to the predominant educational approach described in each study. Studies reporting interventions with multiple educational components were classified according to the dominant or defining component.

Intervention category	n (%)	Interventions/educational approaches included
Clinical exposure	13 (25.0%)	Speciality immersion camp [27], a cadaveric oath and bioethics ceremony [28], early patient-contact programmes [29], service learning [30] [31], a longitudinal patient-partnership programme [32], clinical immersion blocks [33], a transgender-healthcare clinical role-play programme [34], a longitudinal integrated clerkship [35], cadaveric dissection [36], community-based education [37], sonoanatomy skills teaching [38], and a lifestyle-medicine curriculum module [39].
Humanities and arts-based approaches	11 (21.2%)	Healer's art curriculum [40], Holocaust and Medicine curricula [41-43], online visual and literary arts electives [44,45], a comics and graphic-narrative course [46], museum-based pedagogy [47,48], and mask-making, poetry, and comics workshops [49,50].
Reflective and narrative interventions	9 (17.3%)	First-year workshop on core values [51], a dissection-course reflective portfolio and seminar [52], a structured feedback model during clinical assessment [53], a "seven questions" reflective exercise during gross anatomy [54], real-time brief reflections during clinic encounters [55], a reflection-integrated problem-based learning format [56], a pen pal reflective-writing programme [57], a mobile application for reflective peer and mentor interaction [58], and a reflective empathy-portfolio intervention [59].
Mentorship programmes	7 (13.5%)	One-to-one faculty mentoring during clinical courses [60] to longitudinal coaching pairing a faculty coach with a small student group [61], a patient-mentor programme pairing preclinical students with patients living with chronic illness [62], a brief coaching pilot during a sub-internship [63], an online identity-concordant mentor-matching platform [64], a longitudinal faculty-mentoring programme [65], and a structured research-mentoring programme for peer-mentors [66].
Mixed interventions	5 (9.6%)	A 4-year longitudinal course combining workshops, portfolio reflection, and mentoring [67], a leadership and quality-improvement course combining didactic teaching, small groups, and mentored projects [68], a professionalism remediation course combining fieldwork, role-model shadowing, and small-group discussion [69], a scholarly concentration combining mentored dinners, a preceptorship, and skills training [70], and a cluster-randomised crossover trial of resilience and professionalism tutorials [71].
Simulation-based interventions	4 (7.7%)	Simulation-based orientation to medical school [72], a single high-fidelity simulation during an inter-clerkship rotation [73], a single interprofessional simulation session across four universities [74], and an objective structured teaching exercise in which students served as simulated residents [75].
Peer learning	3 (5.8%)	A randomised trial of a single interactive interprofessional learning workshop [26], structured near-peer reflecting teams facilitated by junior residents [76], and digital peer mentoring for first-year students [77].

Seven intervention categories were identified according to the predominant pedagogic mechanism (Table 4). Clinical exposure was the most frequently represented category, followed by humanities and arts-based and reflective/narrative approaches. Mentorship, mixed interventions, simulation, and peer learning were less frequently represented. The distribution indicates that the existing literature has predominantly explored experience-based and reflective approaches to PIF, while fewer studies have evaluated peer-based and simulation approaches.

Theoretical Frameworks and Educational Approaches

A theoretical or conceptual framework was reported in 44 studies (84.6%); frameworks were categorised as explicit PIF models or related educational/social theories (Table 5).

**Table 5:** Theoretical Frameworks in Included Studies (n=52) Authors' analysis of data extracted from the 52 included studies. Framework categories were based on the theoretical or conceptual frameworks reported by the study authors. Frequencies were non-mutually exclusive because individual studies could report multiple frameworks. PIF, professional identity formation

Category	Examples	n (%)
Explicit PIF-specific framework or model	Cruess et al. PIF/socialisation framework (n=6) [33,39,53,56,67,73]; Wald's PIF framework/theory (n=2) [42,43]; Jarvis-Selinger PIF framework (n=1) [39]; Krishna-Pisupati model of PIF (n=1) [66]; socialisation model of PIF (n=1) [30]; patient-centred model of PIF (n=1) [62]; other explicitly PIF-focused frameworks/theoretical conceptualisations (n=4) [32,34,41,63]	15 (28.8%)
Related educational, social, or identity theory	Extended Professional Identity Theory (EPIT) (n=2) [66]; Community of practice or situated learning (n=13) [32,35,38,61,66,69,70,72-77]; Kolb's or other experiential-learning models (n=9) [27,32,33,51,63,67-70]; identity, liminality, or rites-of-passage theory (n=8) [26,29,49,50,58,62,66,74]; socialisation/socialisation theory (n=5) [27,30,37,39,70]; reflective practice/reflective learning models (n=5) [43,46,67,70,71]; social constructivism (n=4) [31,35,53,58]	29 (55.8%)
No framework reported	No framework reported [28,44,45,54,59,60,64,65]	8 (15.4%)

A theoretical or conceptual framework was reported in 44 of the 52 studies (84.6%) (Table 5). PIF-specific frameworks were reported in 15 studies, while 29 used related educational, social, identity, or experiential theories; eight studies did not report a framework. Because several studies reported more than one framework, these categories were not mutually exclusive.

Outcomes and Measurement Approaches

Five outcome domains were identified, with identity development being the predominant domain (32/52, 61.5%). Qualitative-only and mixed-method approaches accounted for most of the measurement strategies. Only three studies (5.8%) used validated quantitative instruments, while 10 (19.2%) used non-validated or locally developed quantitative measures (Table 6).

**Table 6:** Outcome Domains and Measurement Approaches in Included Studies (n=52) Authors' analysis of data extracted from the 52 included studies. Outcome domains were classified according to the primary outcome reported in each study and were therefore mutually exclusive. Measurement categories were assigned according to the dominant measurement approach recorded during data extraction. “Validated” refers to a previously validated instrument or measurement approach; “non-validated or locally developed” refers to study-specific measures without reported validation evidence. PIF, professional identity formation

Domain/measurement characteristic	Studies, n (%)	Examples/details
Outcome domains		
Identity development	32 (61.5%)	Predominant outcome domain, encompassing development of professional identity, role transition, self-concept, professional purpose, and identity-related reflection [27,29,32,33,35-39,44-50,51,53-58,60,61,63,65-67,70,72,73,77]
Professional values	7 (13.5%)	Included values-oriented outcomes involving respect, altruism, ethical awareness, moral reasoning, patient-centred care, and professional responsibility [28,34,41-43,52,62]
Empathy	5 (9.6%)	Included empathy development or its relationship with PIF [30,31,40,55,59]
Belonging	5 (9.6%)	Examined primarily through mentorship, peer learning, and interprofessional/simulation experiences [26,64,74-76]
Behaviour/professionalism	3 (5.8%)	All three studies used mixed-intervention approaches and evaluated behavioural or professionalism-related outcomes [68,69,71]
Measurement approach		
Qualitative methods only	19 (36.5%)	Interviews, reflective narratives, portfolios, qualitative evaluations, thematic analysis, and other qualitative approaches [27,29-31,34,35,37,39,42,43,45,48,52,54,62,66,69,75,76]
Mixed quantitative-qualitative methods	18 (34.6%)	Quantitative measures combined with qualitative data, including reflective/narrative components [26,33,36,38,44,46,47,49,50,55,57,60,63,65,68,71-73]
Quantitative methods using non-validated or locally developed measures	10 (19.2%)	Locally developed questionnaires, surveys, or non-validated quantitative measures addressing identity, reflection, values, belonging, or related constructs [28,32,40,51,53,58,64,67,70,77]
Quantitative methods using validated instruments	3 (5.8%)	Matsuyama et al. [56]: Professional Identity Essay (PIE-J) with the Motivated Strategies for Learning Questionnaire (MSLQ-J); Burford et al. [74]: Readiness for Interprofessional Learning Scale (RIPLS) and social-identity measures; Baseer et al. [59]: Jefferson Scale of Physician Empathy–Student version (JSPE-S) and Professional Identity Questionnaire (PIQ)
Measurement approach not reported/not identifiable	2 (3.8%)	No identifiable outcome-measurement approach was reported in the available study reports [41,61]

Outcome domains were classified according to the primary outcome reported in each study and were therefore mutually exclusive. Measurement categories were similarly assigned according to the dominant measurement approach recorded during data extraction.

Four studies reporting null or near-null effects are summarised in Table 7 [36,56,59,71].

**Table 7:** Studies Reporting Null or Limited Quantitative Findings Authors' analysis of data extracted from the included studies. Studies were included in this table when quantitative analyses reported no statistically significant intervention effect, no significant association, or improvements over time without a differential intervention effect.

Study	Country	Design	Intervention/outcome	Tool used	Main finding
Matsuyama et al. (2021) [56]	Japan	RCT	PIF-oriented reflection/PIF and SRL	PIE-J; MSLQ-J	No significant intervention effect; PIF stages improved over time
Ong et al. (2023) [36]	Singapore	Mixed methods	Cadaveric dissection/PIF	MacLeod-Clark PIF Scale; interviews	No significant association with PIF scores; qualitative findings suggested humanistic learning
Baseer et al. (2024) [59]	Pakistan	RCT	Empathy portfolio/PIF and empathy	PIQ; JSPE-M	No significant between-group difference in PIF or empathy
Byrne et al. (2025) [71]	Ireland	Cluster RCT	Professionalism and resilience tutorials	PSCOM; CSES; BRS	Improvements over time, but no differential tutorial effect

Geographic and Income-Group Distribution

Forty-nine studies (94.2%) originated from high-income countries (HICs), two (3.8%) from LMICs, and one (1.9%) from an upper-middle-income country (UMIC) setting. The LMIC studies were from India [28] and Pakistan [59]; the UMIC study was a multi-country consortium spanning Mexico, Colombia, and Chile [58]. The United States contributed more than one-third of all studies. Humanities/arts, mentorship, mixed, simulation, and peer-learning interventions occurred exclusively in HICs, while the three non-HIC studies involved only clinical exposure [28] and reflective/narrative approaches [58,59].

The Indian study evaluated a cadaveric oath and bioethics programme among 200 MBBS students using a cross-sectional, single-group survey and a locally developed, non-validated questionnaire; no explicit theoretical framework was reported [28]. The upper-middle-income Latin American study evaluated the development and usability of a mobile application designed to facilitate reflective sharing and interactions with peers and mentors across three medical schools; it assessed usability rather than effectiveness in changing PIF [58]. Geographic and temporal distributions are shown in Figure 2.

**Figure 2:**
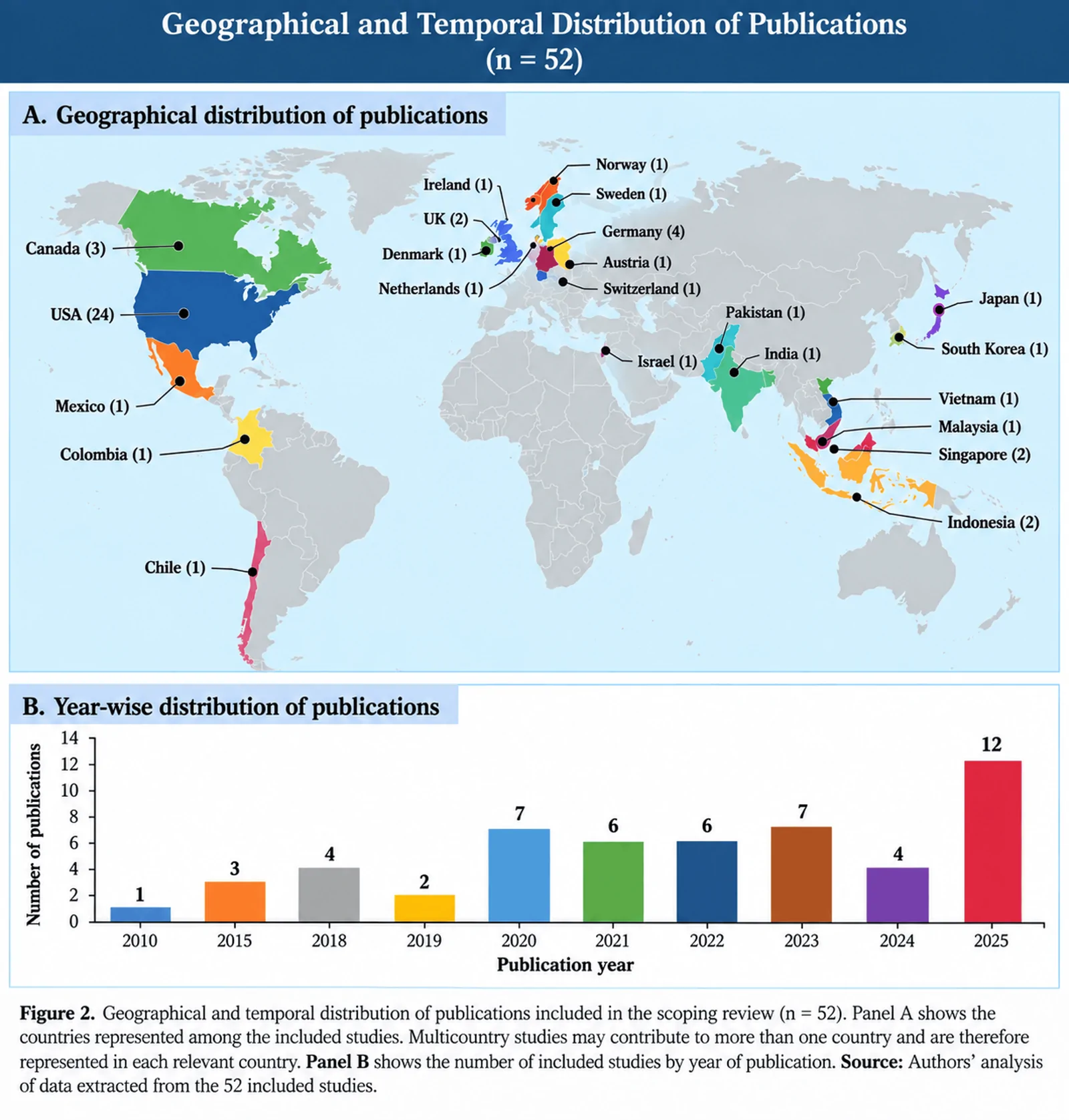
Geographical and Temporal Distribution of Published Studies (n=52) Panel A shows the countries represented among the included studies. Note: Multicountry studies may contribute to more than one country and are therefore represented in each relevant country. Panel B shows the number of included studies by year of publication. Source: This image is an original author-created schematic using Canva Pro (Canva Pty Ltd, Sydney, Australia).

Facilitators, Barriers, and Intersectionality

Authentic clinical or community experience and peer/near-peer learning were the most frequently reported facilitators, followed by supportive environments and opportunities for reflection or debriefing. Common barriers included COVID-19-related disruption, voluntary participation, emotional vulnerability, curriculum time constraints, and short intervention duration (Table 8).

**Table 8:** Reported Facilitators and Barriers to Implementation of PIF Interventions Authors' analysis of data extracted from the 52 included studies. A study could report more than one facilitator or barrier; therefore, categories were not mutually exclusive, and percentages represent the proportion of included studies reporting each factor. PIF, professional identity formation

Domain	Factor	n (%)
Facilitator	Authentic clinical/community experience	21 (40.4)
	Peer/near-peer learning	20 (38.5)
	Supportive environment/reflection/debriefing	18 (34.6)
	Mentoring/faculty engagement	15 (28.8)
	Longitudinal design	10 (19.2)
Barrier	COVID-19 disruption	9 (17.3)
	Selection/voluntary participation	8 (15.4)
	Emotional vulnerability/distress	7 (13.5)
	Curriculum time constraints	5 (9.6)
	Short intervention duration or single-time-point exposure	4 (7.7)
	Cultural/contextual barriers	4 (7.7)
	Measurement limitations	2 (3.8)

Intersectionality was explicitly addressed in four studies (7.7%) [34,49,64,65]. These examined identity-concordant mentorship, personal and religious identities, intersectionality-informed narratives, and gender differences in perceived mentor quality. No study explicitly examined intersecting race, ethnicity, and socioeconomic background in relation to PIF outcomes.

Discussion

Principal Findings

This scoping review mapped the educational interventions used to support PIF in UGME. The evidence suggests that PIF has increasingly moved from being viewed as an implicit consequence of medical training toward an explicit educational objective. However, the field has not yet converged on a coherent approach to designing or evaluating PIF interventions. The major challenge is not simply the diversity of educational approaches, but the limited ability to connect educational activities with theoretically specified mechanisms and measurable developmental outcomes.

Intervention Types and Educational Approaches

The predominance of clinical exposure, humanities and arts, and reflective approaches suggests that PIF education continues to rely heavily on students' direct experiences and opportunities to make meaning from those experiences. This is consistent with established conceptualisations of PIF in which professional identity develops through socialisation, participation, reflection, and interaction with patients, peers, and teachers [9,12]. Previous reviews have similarly identified clinical experiences and longitudinal participation as important contexts for PIF, while highlighting variation in how these experiences are structured [16,78,79]. Thus, the educational value of an intervention may depend not only on its category but also on the opportunities it provides for participation, reflection, feedback, and relationship-building.

At the same time, relatively fewer studies have examined sustained relationship-centred approaches. This is noteworthy because mentoring and role modelling provide opportunities for students to observe professional behaviour, receive feedback, and negotiate their emerging professional identity within authentic relationships [18]. Professional identity therefore develops not only through individual reflection but also through interaction with people and communities that model and reinforce professional norms [9,12,17]. The issue is therefore not that reflection or humanities-based approaches are inappropriate, but that the evidence base gives less attention to how these approaches interact with sustained clinical participation and professional relationships.

The concentration of arts-based interventions within high-income institutions also raises questions about transferability. Their underlying educational principles may be delivered through locally available resources, but the current evidence does not establish whether different modes of delivery produce comparable outcomes. Importantly, this review does not provide evidence that any intervention category is superior to another. Future research should therefore move beyond comparisons between broad intervention labels and examine how specific educational components work together within longitudinal curricula.

Facilitators, Barriers, and Intersectionality

The findings also suggest that the effect and feasibility of PIF interventions cannot be separated from the learning environment in which they occur. Authentic experiences, peer interaction, mentoring, supportive environments, and opportunities for reflection were recurrently identified across the literature. Conversely, limited curriculum time, emotional demands, disruption of clinical exposure, and practical implementation constraints may reduce opportunities for meaningful engagement. Together, these findings reinforce the view that PIF is socially situated and that the effect and feasibility of an intervention depend partly on the learning environment in which it is experienced [12,18].

Attention to students' social identities was considerably less developed. Although some studies considered aspects of identity, few examined how multiple social identities interact with PIF. This is consistent with the broader PIF literature, where personal and sociocultural influences are recognised but remain incompletely investigated [16]. This gap is important because professional identity is formed at the intersection of students' personal identities, social relationships, and professional experiences. Future intervention research should therefore consider whether the same educational experience is experienced similarly by students with different social, cultural, and professional identities.

Theoretical Frameworks

The review demonstrates that the presence of a theoretical framework does not necessarily indicate its meaningful application. The use of PIF-specific models alongside theories of experiential learning, Communities of Practice, socialisation, identity, and reflection reflects the inherently interdisciplinary nature of PIF [9,12,17]. The recent conceptual literature similarly supports the use of complementary theoretical perspectives rather than a single explanatory model.

The more important issue identified by this review is therefore not simply whether a theory was reported, but whether it was used to explain how the intervention was expected to influence identity development. A framework should do more than provide a conceptual description of PIF. It should explain why particular educational activities are expected to influence identity and what changes would be expected if the proposed process occurs. Making this theory-intervention-outcome relationship explicit would improve both intervention development and evaluation. It would also make studies using different theoretical traditions more comparable at the level of their underlying educational assumptions.

Outcomes and Measurement Approaches

The findings indicate that PIF is being evaluated through a wide range of outcomes and measurement approaches. This diversity reflects the multidimensional nature of professional identity but also creates difficulties when findings are compared across studies. Previous reviews have similarly identified substantial variation in PIF assessment, with concerns regarding the consistency and psychometric evidence of available measures [19,80].

The four studies with null or limited quantitative findings illustrate why measurement requires careful interpretation [36,56,59,71]. The absence of a significant quantitative effect did not necessarily indicate an absence of educational value. In one study, qualitative findings suggested humanistic learning despite no significant association between exposure and PIF scores [36]. Other studies involved relatively brief interventions or indirect outcome measures [56,59,71]. Taken together, these findings suggest that intervention duration, outcome selection, and the sensitivity of measurement tools should be considered in relation to the developmental nature of PIF.

The implication is not that qualitative approaches should be replaced by quantitative measures, but that complementary approaches may be particularly appropriate for a construct involving changes in self-understanding, professional values, relationships, and belonging. Longitudinal assessment would also be valuable because immediate changes in reflection or perception may not represent sustained professional identity development. Further work to establish and validate PIF measures across different educational and cultural settings could improve comparability without reducing PIF to a single numerical outcome [19,80,81].

Geographic and Income Setting of the Evidence

The strong concentration of evidence from HICs is an important limitation of the current knowledge base. The limited number of studies from LMIC and UMIC settings does not permit meaningful comparison of interventions across income groups. It does, however, raise questions about the extent to which educational approaches developed in particular institutional and cultural environments can be transferred to other settings.

This geographical concentration is also evident in the broader PIF literature [16,81]. Differences in curriculum structure, student-faculty relationships, clinical exposure, cultural expectations, and available educational resources may influence both the implementation of an intervention and the way students interpret professional identity. Consequently, transfer of an established intervention should not be assumed to represent simple replication. Contextual adaptation and local evaluation are likely to be important priorities for future research.

Strengths and limitations

This review has several strengths. The prospective protocol, systematic searching, structured data charting, and PRISMA-ScR reporting provided a transparent approach to mapping the literature. The review also examined intervention type alongside theoretical framework, outcome, measurement approach, and income setting, allowing gaps to be considered across multiple dimensions of the evidence base.

Several limitations should be considered. As a scoping review, no formal methodological quality appraisal was undertaken, and the findings therefore describe the extent and characteristics of the evidence rather than establishing intervention effectiveness. Restriction to English-language publications may have reduced representation from non-English-speaking settings. Heterogeneity in PIF definitions, intervention designs, outcome measures, and reporting also limited direct comparison. Finally, inductive classification of interventions required judgement, particularly where programmes contained multiple educational components. Although predefined criteria were used, alternative categorisation by another review team is possible. Accordingly, the mapped patterns should be interpreted as descriptive characteristics of the evidence base rather than comparative estimates of intervention effectiveness.

Overall implications

The principal contribution of this review is the integrated mapping of intervention approaches, theoretical grounding, outcomes, measurement, and context within UGME PIF research. The evidence suggests that the field has moved beyond viewing PIF as an implicit consequence of clinical education, but considerable variation remains in how interventions are designed and evaluated.

Three priorities emerge from this evidence: stronger alignment between theory and intervention design, longitudinal and multimethod evaluation, and greater attention to contextual adaptation and representation beyond high-income settings. Together, these priorities may allow PIF research to move from a diverse collection of educational initiatives toward a more cumulative and comparable evidence base.

## Conclusions

This scoping review demonstrates a diverse yet fragmented evidence base for educational interventions that support PIF in UGME. Interventions were concentrated on clinical exposure, the humanities and the arts, and reflective approaches, while theoretical application and validated measurement remained inconsistent. The evidence was also strongly concentrated in high-income settings, limiting understanding of how PIF interventions operate across different educational and cultural contexts.

Future research should strengthen the alignment between theory and intervention design, use validated and contextually appropriate measures, evaluate outcomes longitudinally, and expand research beyond high-income settings. These priorities may support the development of a more comparable, theoretically informed, and context-sensitive evidence base for PIF in UGME.

## Appendices

Appendix

Preferred Reporting Items for Systematic Reviews and Meta-Analyses extension for Scoping Reviews (PRISMA-ScR) checklist

**Table 9:** Preferred Reporting Items for Systematic reviews and Meta-Analyses Extension for Scoping Reviews (PRISMA-ScR) Checklist * Where sources of evidence (see second footnote) are compiled from, such as bibliographic databases, social media platforms, and websites. † A more inclusive/heterogeneous term used to account for the different types of evidence or data sources (e.g., quantitative and/or qualitative research, expert opinion, and policy documents) that may be eligible in a scoping review as opposed to only studies. This is not to be confused with information sources (see first footnote). ‡ The frameworks by Arksey and O’Malley (6) and Levac et al. (7) and the JBI guidance (4 and 5) refer to the process of data extraction in a scoping review as data charting. § The process of systematically examining research evidence to assess its validity, results, and relevance before using it to inform a decision. This term is used for items 12 and 19 instead of "risk of bias" (which is more applicable to systematic reviews of interventions) to include and acknowledge the various sources of evidence that may be used in a scoping review (e.g., quantitative and/or qualitative research, expert opinion, and policy documents). Source: Tricco et al. [24] JBI, Joanna Briggs Institute; PRISMA-ScR, Preferred Reporting Items for Systematic Reviews and Meta-Analyses Extension for Scoping Reviews

Section	Item	PRISMA-ScR checklist item	Reported on page #
TITLE			
Title	1	Identify the report as a scoping review.	p. 1
ABSTRACT			
Structured summary	2	Provide a structured summary that includes (as applicable): background, objectives, eligibility criteria, sources of evidence, charting methods, results, and conclusions that relate to the review questions and objectives.	p. 1
INTRODUCTION			
Rationale	3	Describe the rationale for the review in the context of what is already known. Explain why the review questions/objectives lend themselves to a scoping review approach.	pp. 3-4
Objectives	4	Provide an explicit statement of the questions and objectives being addressed with reference to their key elements (e.g., population or participants, concepts, and context) or other relevant key elements used to conceptualise the review questions and/or objectives.	p. 4
METHODS			
Protocol and registration	5	Indicate whether a review protocol exists; state if and where it can be accessed (e.g., a Web address); and if available, provide registration information, including the registration number.	p. 4
Eligibility criteria	6	Specify characteristics of the sources of evidence used as eligibility criteria (e.g., years considered, language, and publication status), and provide a rationale.	pp. 4-5
Information sources*	7	Describe all information sources in the search (e.g., databases with dates of coverage and contact with authors to identify additional sources), as well as the date the most recent search was executed.	p. 5
Search	8	Present the full electronic search strategy for at least 1 database, including any limits used, such that it could be repeated.	p. 5; Supplementary File S2, pp. 1-2
Selection of sources of evidence†	9	State the process for selecting sources of evidence (i.e., screening and eligibility) included in the scoping review.	pp. 5-6
Data charting process‡	10	Describe the methods of charting data from the included sources of evidence (e.g., calibrated forms or forms that have been tested by the team before their use, and whether data charting was done independently or in duplicate) and any processes for obtaining and confirming data from investigators.	p. 6
Data items	11	List and define all variables for which data were sought and any assumptions and simplifications made.	p. 6
Critical appraisal of individual sources of evidence§	12	If done, provide a rationale for conducting a critical appraisal of included sources of evidence; describe the methods used and how this information was used in any data synthesis (if appropriate).	p. 6; no critical appraisal was undertaken
Synthesis of results	13	Describe the methods of handling and summarising the data that were charted.	p. 6
RESULTS			
Selection of sources of evidence	14	Give numbers of sources of evidence screened, assessed for eligibility, and included in the review, with reasons for exclusions at each stage, ideally using a flow diagram.	p. 7; Figure 1
Characteristics of sources of evidence	15	For each source of evidence, present characteristics for which data were charted and provide the citations.	pp. 7-8; Tables 1-4 and Supplementary Table S1
Critical appraisal within sources of evidence	16	If done, present data on critical appraisal of included sources of evidence (see item 12).	Not applicable; no critical appraisal was undertaken
Results of individual sources of evidence	17	For each included source of evidence, present the relevant data that were charted that relate to the review questions and objectives.	pp. 7-8; Tables 2-6 and Supplementary Table S1
Synthesis of results	18	Summarise and/or present the charting results as they relate to the review questions and objectives.	pp. 7-8; Tables 1-6 and Figure 2
DISCUSSION			
Summary of evidence	19	Summarise the main results (including an overview of concepts, themes, and types of evidence available), link to the review questions and objectives, and consider the relevance to key groups.	pp. 9-13
Limitations	20	Discuss the limitations of the scoping review process.	pp. 12-13
Conclusions	21	Provide a general interpretation of the results with respect to the review questions and objectives, as well as potential implications and/or next steps.	p. 13
FUNDING			
Funding	22	Describe sources of funding for the included sources of evidence, as well as sources of funding for the scoping review. Describe the role of the funders of the scoping review.	p. 13
